# Clinical features and risk factors analysis of diabetes mellitus complicated with Peyronie’s disease

**DOI:** 10.1093/sexmed/qfag012

**Published:** 2026-04-02

**Authors:** Yuanyuan Li, Xuewen Diao, Zulong Wang

**Affiliations:** Department of Andrology, The First Affiliated Hospital of Henan University of Traditional Chinese Medicine, Zhengzhou 450000, Henan, China; The First Clinical Medical College of Henan University of Traditional Chinese Medicine, Zhengzhou 450000, Henan, China; Department of Andrology, The First Affiliated Hospital of Henan University of Traditional Chinese Medicine, Zhengzhou 450000, Henan, China; The First Clinical Medical College of Henan University of Traditional Chinese Medicine, Zhengzhou 450000, Henan, China; Department of Andrology, The First Affiliated Hospital of Henan University of Traditional Chinese Medicine, Zhengzhou 450000, Henan, China

**Keywords:** Peyronie’s disease, diabetes mellitus, risk factors, retrospective studies, propensity score matching

## Abstract

**Background:**

The association between Diabetes Mellitus (DM) and Peyronie’s disease (PD), as well as the impact of DM on PD phenotypes, remains not fully elucidated.

**Aim:**

To investigate the association between PD and DM and to assess the impact of DM on the clinical phenotype of PD.

**Methods:**

This case–control study retrospectively enrolled 136 PD patients from Department of Andrology, The First Affiliated Hospital of Henan University of Chinese Medicine (December 2019 to May 2025) as the case group. Two control groups were established concurrently: andrology patients without PD and healthy examinees. To control for confounding bias, propensity score matching (PSM) was used to balance baseline characteristics. Logistic regression analysis was employed to determine the independent association between DM and PD. Furthermore, clinical characteristics were compared between PD patients with and without DM.

**Outcomes:**

This study evaluate the correlation between DM and PD, as well as the clinical manifestations of their comorbidity.

**Results:**

① The prevalence of DM and the proportion of related risk factors were significantly higher in the case group (*P* < .05). ② After PSM, logistic regression confirmed an independent association between DM and PD (Model 1: OR = 2.815, 95% CI: 1.376-5.989; Model 2: OR = 3.436, 95% CI: 1.823-6.709). ③ Compared to the non-DM subgroup, PD patients in the DM subgroup had a greater number of plaques, higher plaque elasticity, and a larger curvature angle, but reported lower pain scores (all *P* < .05).

**Clinical Implications:**

This finding suggests that clinicians should be vigilant for occult yet structurally severe PD in diabetic patients and consider implementing multidisciplinary management strategies.

**Strengths and Limitations:**

The strengths of this study lie in its relatively large sample size of PD patients within the field of andrological rare diseases, the dual-control design, and the robust PSM analysis conducted. Limitations include its retrospective, single-center design and the potential for residual confounding factors.

**Conclusion:**

DM is independently associated with PD and is linked to a distinct clinical phenotype characterized by more severe fibrosis (more plaques, greater curvature) but diminished pain perception.

## Introduction

Peyronie’s disease (PD) is a urological condition characterized by fibrotic plaques of the tunica albuginea, painful erection, penile curvature, and erectile dysfunction.^1^^,^^2^ Although not life-threatening, the disease significantly impacts patients’ quality of life, particularly sexual health and psychological well-being, often leading to anxiety, depression, and severe relationship distress.^3^^,^^4^ Its exact etiology remains incompletely understood, and current clinical treatments are limited with suboptimal efficacy. Consequently, identifying modifiable risk factors is crucial for improving patient outcomes. Although previous studies^5–8^ have suggested associations of PD with age, penile trauma, and genetic predisposition, research in this field, particularly within Chinese populations, remains challenging. This is primarily due to the relatively low incidence of PD, which has resulted in most published clinical studies having limited sample sizes, often consisting of case reports or series analyzes involving only dozens of patients.^9^^,^^10^ The constraints of these small-sample studies make it difficult for researchers to perform adequate subgroup analyzes or control for complex confounding factors. This, in turn, has led to existing evidence on the association between important metabolic factors like Diabetes Mellitus (DM) and PD being both weak and inconsistent, greatly limiting a comprehensive understanding of the risk profile of Chinese PD patients. To overcome these limitations, this study retrospectively established a substantial single-center clinical cohort of PD (*n* = 136). For this rare condition, this sample size provides a solid foundation for addressing the insufficient statistical power of previous small-scale studies. Leveraging this scarce resource, we innovatively adopted a rigorous case–control design incorporating dual control groups (andrology non-PD patients and healthy examinees combined with Propensity Score Matching (PSM). This methodology aims to minimize selection bias and confounding effects to answer the following central question: Is there a correlation between DM and PD, and what are the clinical manifestations when the two diseases coexist?

## Methods

### General data

Medical records of patients with PD (hereinafter referred to as Group A), general andrology outpatients without PD (hereinafter referred to as Group B), and healthy examinees from the health examination center (hereinafter referred to as Group C) were selected from Department of Andrology, The First Affiliated Hospital of Henan University of Chinese Medicine between December 1, 2019, and May 31, 2025.

### Diagnostic criteria

#### Diagnostic criteria for PD

The diagnostic criteria were established with reference to the *PD: AUA Guideline*.^11^ A diagnosis was made if either of the following was present: palpable nodules or plaques in the tunica albuginea or corpora cavernosa identified by physical examination, OR detection of penile nodules or plaques by penile ultrasonography.

#### *Diagnostic criteria for DM* ^12^

1) Documented history of DM;
2) History of regular use of hypoglycemic agents or subcutaneous insulin administration;
3) Meeting any one of the following laboratory criteria:Fasting venous plasma glucose ≥7.0 mmol/L, or 2-hour venous plasma glucose during OGTT ≥11.1 mmol/L, or HbA1c ≥6.5%, or Random venous plasma glucose ≥11.1 mmol/L.

The presence of any one of the above three criteria confirms the diagnosis of Dm.

### Inclusion criteria

Group A: Patients who met the diagnostic criteria for PD were included.

### Exclusion criteria

Group A: Patients were excluded if they had penile plaques or masses considered or confirmed to be caused by tumor or malignancy, or penile plaques due to syphilis infection.

Groups A, B, and C: Individuals with incomplete medical history records regarding DM were excluded.

### Research methods

#### Variable definitions

Potential risk factors for PD reported in the literature and observed clinically were included. The collected clinical data comprised: age, smoking status, alcohol consumption, sleep deprivation, DM, hypertension, coronary heart disease, hyperlipidemia, history of penile trauma, history of masturbation, and history of immune system diseases.

1) Smoking status was categorized as:

Never-smoker: Lifelong non-smoker or having smoked <100 cigarettes in total.

Former smoker: Having smoked ≥100 cigarettes in total but having quit for ≥1 month at the time of survey.

Current smoker: Having smoked ≥100 cigarettes in total and still smoking at the time of survey.

2) Alcohol consumption was categorized as:

Never-drinker: Having consumed <12 standard alcoholic drinks in one’s lifetime.

Former drinker: Had a history of alcohol consumption but abstained in the year prior to the survey.

Current drinker: Consumed alcohol within the year prior to the survey.

3) DM: Diagnostic criteria referenced section 1.2.2.
4) Hypertension: Defined as systolic blood pressure ≥ 140 mmHg and/or diastolic blood pressure ≥ 90 mmHg.
5) Hyperlipidemia: Diagnosed if any one or more of the following four fasting lipid criteria were met: (1) Serum total cholesterol (TC) >5.2 mmol/L; (2) Triglycerides (TG) >1.7 mmol/L; (3) High-density lipoprotein cholesterol (HDL-C) <1.0 mmol/L; (4) Low-density lipoprotein cholesterol (LDL-C) >3.4 mmol/L.
6) History of penile trauma: Defined as patient-reported injury to the penis caused by external force.
7) History of immune system diseases: Defined as the presence of any one of the following: rheumatoid arthritis, systemic lupus erythematosus, ankylosing spondylitis, Sjögren’s syndrome, or congenital immunodeficiency.
8) Coronary heart disease (CHD): Defined as a previous diagnosis of CHD.

#### Data imputation

An evaluation of missing data in selected baseline covariates was conducted. The variables exhibiting missing values included history of alcohol consumption (5.1% missing), and history of immune system diseases (4.4% missing). To mitigate potential selection bias and loss of statistical power associated with complete-case analysis, multiple imputation was employed. Under the missing at random assumption, we used the Fully Conditional Specification method, incorporating all other study variables as predictors, to generate 5 complete datasets. The imputed datasets were utilized in all subsequent analyzes.

#### Logistic regression

A double case–control study design was employed. After balancing confounding factors using the PSM method, logistic regression analysis was conducted with diabetes status as the independent variable and the incidence of PD as the dependent variable to clarify their correlation.

#### Intergroup comparison

Concurrently, patients in Group A were stratified into a diabetes group and a non-diabetes group based on the presence or absence of diabetes. Clinical data collected from Group A included age, disease duration, number of penile plaques, location of plaques, plaque size, mean plaque elasticity measured by the shear wave elasticity software equipped on the Mindray Resona series ultrasound systems (The manufacturer is located in Shenzhen City, Guangdong Province, China.), pain score (assessed using the Visual Analog Scale [VAS]^13^, where 0 indicates no pain and 10 indicates severe pain), and curvature angle (measured as the angle of deviation from the normal axis during erection). Differences in the aforementioned indicators were then analyzed between these two subgroups.

### Statistical analysis

Statistical analyzes were performed using IBM SPSS Statistics (Version 29.0; IBM Corp., Armonk, NY, USA). Continuous variables were first tested for normality. Data conforming to a normal distribution are presented as mean ± standard deviation *($\bar{{x}} $ ± s)* and were compared between groups using the independent samples *t*-test. Data not conforming to a normal distribution were analyzed using the Mann–Whitney U test. Categorical data are presented as frequencies and percentages, and comparisons between groups were made using the chi-square *(χ^2^)* test. Multivariate analysis was conducted using a logistic regression model. A two-tailed *P*-value of less than .05 was considered statistically significant.

## Results

### Association between DM and PD

#### Comparison of general characteristics

A total of 136 participants were ultimately included in each of Groups A, B, and C. Compared to Group B, Group A (PD patients) were significantly older and had a significantly higher prevalence of DM, smoking, and hyperlipidemia (all *P* < .05; eg, Table 1). When compared to Group C (healthy controls from the health examination center), Group A similarly demonstrated a higher mean age and higher proportions of individuals with DM, a history of penile trauma, smoking, alcohol consumption, and hyperlipidemia (all *P* < .05; eg, Table 2).

**Table 1:** General information of patients in groups A and B.

Variable	Level	Group B^a^	Group A^b^	*P*	SMD
Age (years)		42.191 ± 11.286	48.11 ± 9.638	<.001^^*^^	0.564
Diabetes mellitus (%)	No	111 (81.62)	83 (61.03)	<.001^^*^^	0.468
	Yes	25 (18.38)	53 (38.97)		
Penile trauma (%)	No	134 (98.53)	127 (93.38)	.065	0.264
	Yes	2 (1.47)	9 (6.62)		
Smoking (%)	Never-smoker	23 (16.91)	24 (17.65)	.016^^*^^	0.354
	Former smoker	14 (10.29)	31 (22.79)		
	Current smoker	99 (72.79)	81 (59.56)		
Alcohol consumption (%)	Never-drinker	19 (13.97)	24 (17.65)	.151	0.237
	Former drinker	21 (15.44)	31 (22.79)		
	Current drinker	96 (70.59)	81 (59.56)		
Hypertension (%)	No	109 (80.15)	113 (83.09)	.639	0.076
	Yes	27 (19.85)	23 (16.91)		
Hyperlipidemia (%)	No	121 (88.97)	136 (100.00)	<.001^^*^^	0.498
	Yes	15 (11.03)	0 (0.00)		
Immune System Disorders (%)	No	130 (95.59)	124 (91.18)	.223	0.178
	Yes	6 (4.41)	12 (8.82)		
Coronary heart disease (%)	No	131 (96.32)	129 (94.85)	.768	0.072
	Yes	5 (3.68)	7 (5.15)		

^^*^^Between-group differences are statistically significant. ^a^Group B included 136 cases. ^b^Group A included 136 cases.

**Table 2:** General information of patients in groups A and C.

Variable	Level	Group C^a^	Group A^b^	*P*	SMD
Age (years)		45.544 ± 11.208	48.11 ± 9.638	.044^^*^^	0.246
Diabetes mellitus (%)	No	116 (85.29)	83 (61.03)	<.001^^*^^	0.569
	Yes	20 (14.71)	53 (38.97)		
Penile trauma (%)	No	135 (99.26)	127 (93.38)	.024^^*^^	0.316
	Yes	1 (0.74)	9 (6.62)		
Smoking (%)	Never-smoker	39 (28.68)	19 (13.97)	.006^^*^^	0.397
	Former smoker	27 (19.85)	24 (17.65)		
	Current smoker	70 (51.47)	93 (68.38)		
Alcohol consumption (%)	Never-drinker	35 (25.74)	12 (8.82)	.001^^*^^	0.465
	Former drinker	28 (20.59)	30 (22.06)		
	Current drinker	73 (53.68)	94 (69.12)		
Hypertension (%)	No	113 (83.09)	105 (77.21)	.287	0.148
	Yes	23 (16.91)	31 (22.79)		
Hyperlipidemia (%)	No	125 (91.91)	110 (80.88)	.013^^*^^	0.326
	Yes	11 (8.09)	26 (19.12)		
Immune System Disorders (%)	No	130 (97.06)	124 (91.18)	.071	0.252
	Yes	4 (2.94)	12 (8.82)		
Coronary heart disease (%)	No	131 (98.53)	129 (94.85)	.175	0.207
	Yes	2(1.47)	7 (5.15)		

^^*^^Between-group differences are statistically significant.^a^Group C included 136 cases. ^b^Group A included 136 cases.

#### Univariate logistic regression analysis for PD after PSM

To control for potential confounding variables, PSM was performed using the nearest-neighbor method with a caliper width of 0.02 and a 1:1 matching ratio. Covariates included age, history of penile trauma, smoking history, alcohol consumption history, and history of hyperlipidemia. After PSM, 81 matched pairs were successfully generated for the comparison between Group A and Group B, and 106 matched pairs for the comparison between Group A and Group C. In both of these matched cohorts, the prevalence of DM remained significantly higher in the PD group (*P* < .05). Subsequent logistic regression analysis confirmed an independent positive association between DM and PD (Model 1: OR = 2.815, 95% CI: 1.376-5.989, *P* < .05; Model 2: OR = 3.436, 95% CI: 1.823-6.709, *P* < .05; eg, Table 3).

**Table 3:** Logistic regression analysis was performed between group A and group B and group C.

Variable	Group comparisons	*P*	OR	95%CI	
				Lower limit	Upper limit
Diabetes mellitus	Groups A and B^a^	.006	2.815	1.376	5.989
	Groups A and C^b^	.0002	3.436	1.823	6.709

a The sample size of group A and group B after PSM was 81 cases each. ^b^The sample size of group A and group C after PSM was 106 cases each.

### **Association between diabetes and severity of** PD

#### Baseline characteristics of group A patients

When patients in Group A were stratified into subgroups based on the presence or absence of DM, those in the diabetes subgroup were significantly older than those in the non-diabetes subgroup (*P* < .05). However, no statistically significant difference was observed in terms of disease duration between the two subgroups (*P* > .05; eg, Table 4).

**Table 4:** Comparison of clinical features between diabetic and non-diabetic groups.

Variable	Diabetes group^a^	Non-diabetic group^b^
Age (years)	50.65 ± 8.71	46.94 ± 9.86^^*^^
Duration of illness (months)	15.95 ± 10.35	12.57 ± 11.20
Number of penile plaques (pcs)	1.91 ± 1.02	1.29 ± 0.60^^*^^
Plaque elasticity (Kpa)	80.25 ± 18.75	65.15 ± 23.17^^*^^
Plaque size (mm^2^)	153.42 ± 43.25	145.57 ± 38.95
Pain Scores (points)	1.35 ± 0.78	3.47 ± 2.26^^*^^
Penile curvature (°)	24.65 ± 16.35	17.89 ± 9.54^^*^^

Values are presented as mean ± standard deviation. ^a^53 cases in the diabetes group, ^b^83 cases in the non-diabetic group, ^^*^^compared with the diabetic group.

#### Comparison of PD-related indicators in group A

The diabetes subgroup had a significantly greater number of penile plaques and a significantly higher mean plaque elasticity compared to the non-diabetes subgroup. However, no statistically significant difference was found in plaque size between the two groups. (eg, Table 4).

#### Comparison of pain score and curvature angle in group A

Patients in the diabetes subgroup reported a significantly lower pain score compared to those in the non-diabetes subgroup. Regarding penile curvature, the diabetes subgroup exhibited a significantly greater curvature angle, indicating more severe deformity (eg, Table 4).

## Discussion

Utilizing a rigorous dual-control design and PSM analysis, this study clearly demonstrates two key findings. First, an independent positive association exists between DM and PD. Even after controlling for confounding factors such as age, history of penile trauma, smoking, alcohol consumption, and hyperlipidemia, the risk of developing PD was significantly higher in the diabetes group (OR = 2.815 and 3.436, respectively). Second, patients with PD in the diabetes subgroup presented a distinct clinical phenotype characterized by a “pain-curvature dissociation.” Specifically, compared to non-diabetic PD patients, diabetic PD patients showed no difference in disease duration but exhibited more severe structural pathology, including a greater number of penile plaques, higher mean plaque elasticity, and a larger curvature angle, while simultaneously reporting significantly less pain.

DM^14^ a common chronic metabolic disorder, is known for its systemic complications affecting multiple organ systems. Recent studies have suggested a potential association between DM and PD,^15^^,^^16^ implicating a complex interplay of factors such as metabolic disturbances, oxidative stress, and inflammatory responses. The more severe structural phenotype observed in the PD patients with diabetes in this study is likely attributable to diabetes-driven pro-fibrotic mechanisms. Research indicates^17^ that chronic hyperglycemia in diabetic patients leads to the accumulation of advanced glycation end products (AGEs) in penile cavernous tissues and enhances oxidative stress, generating increased reactive oxygen species (ROS), which subsequently accelerates the tissue fibrosis process. AGEs can directly cross-link collagen proteins, reducing tissue elasticity.^18^ Concurrently, ROS damage DNA, proteins, and lipids, causing cellular dysfunction and activating signaling pathways such as NF-κB, which induces the release of pro-inflammatory factors^19^ and triggers a sustained inflammatory response. These mechanisms collectively promote abnormal fibrosis of the tunica albuginea and plaque formation. Animal studies have confirmed significantly higher AGEs content in diabetic penile tissues compared to non-diabetic controls.^20^ Other studies have reported significantly elevated levels of Matrix Metalloproteinases (MMPs) in diabetic patients,^21^ and an imbalance between MMPs and their Tissue Inhibitors of Metalloproteinases may also contribute to plaque formation.^15^ From a genetic perspective, which may be related to the WNT-2 gene and TGF-β1 gene, a study involving 57 patients diagnosed with PD showed that in PD patients comorbid with diabetes, the homozygous expression of the WNT-2 gene was significantly upregulated, suggesting that this gene may serve as a key molecular hub linking the pathophysiological processes of the two diseases.^22^ Studies have also indicated that the TGF-β1 gene can influence the fibrotic process. TGF-β1 serves as a central pro-fibrotic factor, capable of activating fibroblasts, promoting collagen synthesis, and inhibiting its degradation.^23^ The clinical observations in this study—specifically, a greater number of plaques and higher plaque elasticity in the diabetes subgroup—represent the clinical manifestation of this pathological process. This suggests that diabetes may accelerate and exacerbate local fibrosis, leading to more severe structural damage in PD.

It is particularly noteworthy that despite exhibiting more severe fibrosis and greater penile curvature, diabetic PD patients reported significantly lower pain scores. This seemingly paradoxical phenomenon is highly likely associated with diabetic peripheral neuropathy (DPN).^24^ DPN can impair the function of sensory nerve fibers,^25^ reducing patients’ pain sensitivity and thereby masking pain, a typical symptom of PD in the acute phase. This “silent progression,” characterized by significant structural damage but absent or diminished pain perception, represents a crucial clinical feature of PD comorbid with diabetes. It may lead to delayed medical consultation by patients and make early detection difficult for physicians during routine examinations.

## Conclusions

In summary, the value of this study lies in utilizing a substantial PD cohort from a Chinese population to not only confirm the independent association between DM and PD but, more importantly, to reveal a distinct PD clinical phenotype shaped by DM. This finding updates our understanding of the PD clinical spectrum and carries direct clin ical implications: for patients with DM, andrologists should be vigilant for the possibility of occult, yet structurally more severe, PD. For male diabetic patients with longer disease duration or poor glycemic control, incorporating penile palpation and routine examination into general check-ups could aid in early diagnosis. Future research should focus on elucidating the mediating role of neuropathy in this process and exploring multidisciplinary management strategies.

This study has several limitations. Its single-center, retrospective design and single source of participants may introduce selection bias. Furthermore, inherent to retrospective studies, numerous data points were missing for some cases; for instance, histories of masturbation and sleep deprivation could not be fully ascertained. To address this, data imputation methods were employed to mitigate bias from missing data and preserve statistical power. Although PSM was performed, it remains difficult to entirely eliminate all unmeasured or unknown residual confounding factors. Future multicenter, prospective studies with improved data collection protocols are needed to more accurately evaluate the relationship between DM and PD.

## References

[1] Feyisetan O . Peyronie's disease: a brief overview. Cureus. 2023;15(4):e37037. 10.7759/cureus.37037 37143639 PMC10153789

[2] Di Maida F, Cito G, Lambertini L, et al. The natural history of Peyronie's disease. World J Mens Health. 2021;39(3):399–405. 10.5534/wjmh.200065 32648381 PMC8255406

[3] Paulis G, De Giorgio G, Paulis A. Clinical presentation of Peyronie's disease: a retrospective study of 564 cases. Diagnostics (Basel). 2024;14(11):1125. 10.3390/diagnostics14111125 38893650 PMC11172383

[4] Nelson CJ, Mulhall JP. Psychological impact of Peyronie's disease: a review. J Sex Med. 2012;10(3):653–660. 10.1111/j.1743-6109.2012.02999.x 23153101

[5] Schwarzer U, Sommer F, Klotz T, Braun M, Reifenrath B, Engelmann U. The prevalence of Peyronie's disease: results of a large survey. BJU Int. 2001;88(7):727–730. 10.1046/j.1464-4096.2001.02436.x 11890244

[6] Jarow JP, Lowe FC. Penile trauma: an etiologic factor in Peyronie's disease and erectile dysfunction. J Urology. 1997;158(4):1388–1390. 10.1016/S0022-5347(01)64222-8 9302127

[7] Usta MF, Bivalacqua TJ, Jabren GW, et al. Relationship between the severity of penile curvature and the presence of comorbidities in men with Peyronie's disease. J UROLOGY. 2004;171(2 Pt 1):775–779. 10.1097/01.ju.0000097498.34847.7c 14713809

[8] Reddy AG, Dai MC, Song JJ, Pierce HM, Patel SR, Lipshultz LI. Peyronie's disease: an outcomes-based guide to non-surgical and novel treatment modalities. Res Rep Urol. 2023;15:55. 10.2147/RRU.S278796 36756281 PMC9901485

[9] Jin DC, Luo Y, Wang P, et al. Microscopic electric grinding of tunica albuginea plaques and graft repair for Peyronie's disease: a report of 21 cases. J Army Med Univ. 2024;46(11):1291–1297. 10.16016/j.2097-0927.202309044

[10] Li ZG, Zang GH, Hao L, Liu YB, Niu ZJ, Gu XJ. Plaque excision and autologous tunica vaginalis grafting for Peyronie's disease. Natl J Androl. 2018;24(1):55–58. 10.13263/j.cnki.nja.2018.01.010 30157361

[11] Nehra A, Alterowitz R, Culkin DJ, et al. Peyronie's disease: AUA guideline. J UROLOGY. 2015;194(3):745–753. 10.1016/j.juro.2015.05.098 PMC5027990 26066402

[12] American Diabetes Association . (2) classification and diagnosis of diabetes. Diabetes Care. 2015;38(Suppl):S8–S16. 10.2337/dc15-S005 25537714

[13] Wikström L, Eriksson K, Fridlund B, Nilsson M, Årestedt K, Broström A. The clinical applicability of a daily summary of patients' self-reported postoperative pain-a repeated measure analysis. J Clin Nurs. 2017;26(23-24):4675–4684. 10.1111/jocn.13818 28334471

[14] Erdin Z, Çifci A. Type 2 diabetes mellitus: current diagnosis and treatment. JTPM. 2023;2(3):118–125. 10.51271/JTPM-0053

[15] Gelbard MK, Rosenbloom J. Fibroproliferative disorders and diabetes: understanding the pathophysiologic relationship between Peyronie's disease, Dupuytren disease and diabetes. Endocrinol. Diabetes Metab. 2021;4(2):e00195. 10.1002/edm2.195 PMC8029506 33855203

[16] Gianazza S, Belladelli F, Leni R, et al. Peyronie's disease development and management in diabetic men. Andrology-Us. 2023;11(2):372–378. 10.1111/andr.13224 35771713

[17] Ban CR, Twigg SM. Fibrosis in diabetes complications: pathogenic mechanisms and circulating and urinary markers. Vasc Health Risk Man. 2008;4(3):575–596. 10.2147/VHRM.S1991 PMC2515418 18827908

[18] Cai XB, Fan JG, Tian LY, Xu ZJ. Effects of glycosylation end products on presynthetic collagen and fibrogenic cytokines in activated hepatic stellate cells. J Clinical Hepatobiliary Dis. 2010;26(04):403–406.

[19] McGowan TA, Dunn SR, Falkner B, Sharma K. Stimulation of urinary TGF-beta and isoprostanes in response to hyperglycemia in humans. Clin J Am Soc Nephro. 2006;1(2):263–268. 10.2215/CJN.00990905 17699215

[20] Yang C, Ding YX, Kong CZ, Liu XW, Zhang GJ. Expression analysis of glycosylation end product and inducible nitric oxide synthase in penile tissue of diabetic rats. China Contemporary Med. 2015;22(06):8–12.

[21] Derosa G, D'Angelo A, Tinelli C, et al. Evaluation of metalloproteinase 2 and 9 levels and their inhibitors in diabetic and healthy subjects. Diabetes Metab. 2007;33(2):129–134. 10.1016/j.diabet.2006.11.008 17320450

[22] Toprak E, Keskin E, Gezdirici A, Otunctemur A, Canat H. Evaluation of similar genetic pathophysiology underlying diabetes mellitus and Peyronie’s disease: *WNT-2* and *TGF Beta-1* genes. Haseki Tip Bul. 2025;62(2):92–96. 10.4274/haseki.galenos.2024.9534

[23] Patel DP, Christensen MB, Hotaling JM, Pastuszak AW. A review of inflammation and fibrosis: implications for the pathogenesis of Peyronie's disease. World J Urol. 2019;38(2):253–261. 10.1007/s00345-019-02815-6 31190155 PMC7333524

[24] Yang K, Wang Y, Li YW, et al. Progress in the treatment of diabetic peripheral neuropathy. Biomed Pharmacother. 2022;148:112717. 10.1016/j.biopha.2022.112717 35193039

[25] Yu Y . Gold standard for diagnosis of DPN. Front Endocrinol (Lausanne). 2021;12:719356. 10.3389/fendo.2021.719356 34764937 PMC8576350

